# P-1572. Epidemiology and Risk Factors for Altered Mental Status Induced by Urinary Tract Infections Amongst Elderly Hospitalized Patients

**DOI:** 10.1093/ofid/ofae631.1739

**Published:** 2025-01-29

**Authors:** Rebecca Cino, William Lindley, Joseph Reilly, Thomas Lodise, Manish Trivedi

**Affiliations:** AtlantiCare Regional Medical Center, Pomona, New Jersey; AtlantiCare Regional Medical Center, Pomona, New Jersey; AtlantiCare Regional Medical Center, Pomona, New Jersey; Albany College of Pharmacy and Health Sciences, Albany, New York; AtlantiCare Regional Medical Center, Pomona, New Jersey

## Abstract

**Background:**

Elderly patients with urinary tract infections (UTIs) often present with atypical symptoms including altered mental status (AMS). Although UTI-induced AMS is typically reversible, these patients often experience complications and a longer hospital stay. There is a paucity of data on the incidence and risk factors for UTI-induced AMS in elderly patients. The purpose of this study was to describe the epidemiology and risk factors for AMS in elderly patients hospitalized with UTIs.

**Table 1.**
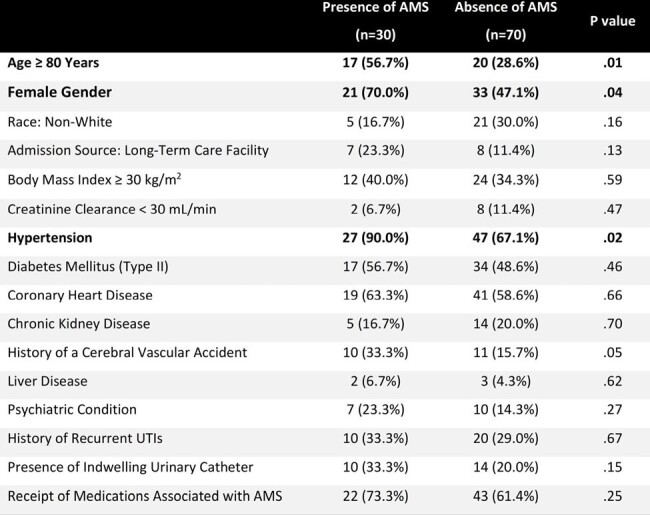
Bivariate Comparison of Baseline Characteristics between Patients with and without UTI-induced Altered Mental Status (AMS)

χ2 test, Student’s t-test, or Fisher’s exact test for variable comparisons.

**Methods:**

A retrospective study of hospitalized patients ≥ 70 years of age with a UTI at AtlantiCare Regional Medical Center between 1/2023-12/2023 was performed. Subjects with UTI were categorized in 2 groups, those with new onset UTI-induced AMS and those without AMS. Admitting providers determined the presence of AMS defined as new onset delirium or a significant change in mental status from baseline. Patients were excluded if they displayed evidence of AMS prior to their UTI or had a new comorbid condition at admission that could precipitate AMS. Data collection included patient demographics, body mass index, weight, admission source, comorbid conditions, presence of urinary instrumentation, and concurrent medications. Bivariate analyses were completed for baseline characteristics between patients with and without UTI-induced AMS. Log-binomial regression was performed to determine factors independently associated with the presence of UTI-induced AMS.

**Table 2.**
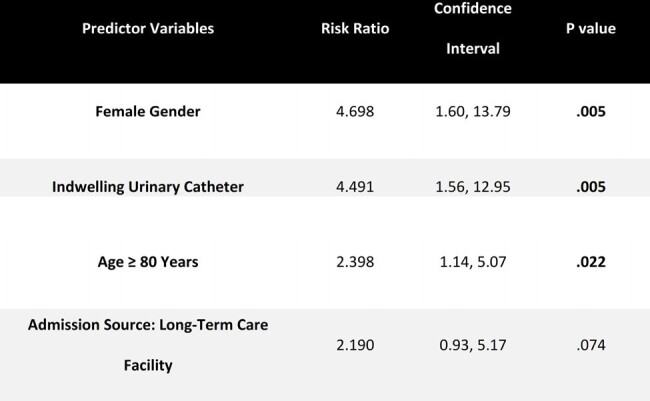
Baseline Characteristics Associated with UTI-induced Altered Mental Status (AMS) in the Log-Binomial Regression Analysis

**Results:**

In total, 100 patients met the study criteria. The mean (SD) age was 74.8 (4.8) years, 54% were female, 15% were admitted from a long-term care facility, and 24% had an indwelling urinary catheter. Comparison of baseline characteristics between patients with and without UTI-induced AMS is shown in **Table 1**. In the log-binomial regression, female gender [risk ratio (RR) of 4.7, 95% CI: 1.6-13.8], presence of a urinary catheter (RR of 4.5, 95% CI: 1.6-13.0), and age ≥ 80 years (RR of 2.4, 95% CI: 1.1-5.1) were independently associated with UTI-induced AMS (**Table 2**).

**Conclusion:**

Identified risk factors for UTI-induced AMS included female gender, presence of an indwelling urinary catheter, and age 80 years or greater. Further studies are warranted to determine the clinical implications and significance of these potential risk factors.

**Disclosures:**

**Joseph Reilly, B.S., Pharm.D.**, Melinta Therapeutics: Advisor/Consultant **Thomas Lodise, Jr., Pharm.D., PhD**, MERCK: Advisor/Consultant

